# Respiratory oxygen consumption in the seagrass *Zostera marina* varies on a diel basis and is partly affected by light

**DOI:** 10.1007/s00227-017-3168-z

**Published:** 2017-05-27

**Authors:** Lina M. Rasmusson, Chiara Lauritano, Gabriele Procaccini, Martin Gullström, Pimchanok Buapet, Mats Björk

**Affiliations:** 1 Seagrass Ecology and Physiology Research Group, Department of Ecology, Environment and Plant Sciences, Stockholm University, SE-106 91 Stockholm, Sweden; 2 Department of Integrative Marine Ecology, Stazione Zoologica Anton Dohrn, Villa Comunale, 80121 Naples, Italy; 3 Department of Biology, Faculty of Science, Prince of Songkla University, Hat Yai, Songkhla 90112 Thailand

## Abstract

The seagrass *Zostera marina* is an important marine ecosystem engineer, greatly influencing oxygen and carbon fluctuations in temperate coastal areas. Although photosynthetically driven gas fluxes are well studied, the impact of the plant’s mitochondrial respiration on overall CO_2_ and O_2_ fluxes in marine vegetated areas is not yet understood. Likewise, the gene expression in relation to the respiratory pathway has not been well analyzed in seagrasses. This study uses a combined approach, studying respiratory oxygen consumption rates in darkness simultaneously with changes in gene expression, with the aim of examining how respiratory oxygen consumption fluctuates on a diel basis. Measurements were first made in a field study where samples were taken directly from the ocean to the laboratory for estimations of respiratory rates. This was followed by a laboratory study where measurements of respiration and expression of genes known to be involved in mitochondrial respiration were conducted for 5 days under light conditions mimicking natural summer light (i.e., 15 h of light and 9 h of darkness), followed by 3 days of constant darkness to detect the presence of a potential circadian clock. In the field study, there was a clear diel variation in respiratory oxygen consumption with the highest rates in the late evening and at night (0.766 and 0.869 µmol O_2_ m^−2^ s^−1^, respectively). These repetitive diel patterns were not seen in the laboratory, where water conditions (temperature, pH, and oxygen) showed minor fluctuations and only light varied. The gene expression analysis did not give clear evidence on drivers behind the respiratory fluxes; however, expression levels of the selected genes generally increased when the seagrass was kept in constant darkness. While light may influence mitochondrial respiratory fluxes, it appears that other environmental factors (e.g., temperature, pH, or oxygen) could be of significance too. As seagrasses substantially alter the proportions of both oxygen and inorganic carbon in the water column and respiration is a great driver of these alterations, we propose that acknowledging the presence of respiratory fluctuations in nature should be considered when estimating coastal carbon budgets. As dark respiration in field at midnight was approximately doubled from that of midday, great over-, or underestimations of the respiratory carbon dioxide release from seagrasses could be made if values are just obtained at one specific time point and considered constant.

**Electronic supplementary material:**

The online version of this article (doi:10.1007/s00227-017-3168-z) contains supplementary material, which is available to authorized users.

## Introduction

*Zostera marina* L. (eelgrass) is the most abundant seagrass species in temperate coastal areas of the northern hemisphere (den Hartog and Kuo 2006) with a substantial yet declining coverage (e.g., Baden et al. 2003; Nyqvist et al. 2009). Seagrasses establish habitats of high ecological and economic value. Commercially important fish and invertebrate species find feeding and nursery grounds in seagrass meadows, the leaves can enhance sedimentation rates of particles in the water column, and belowground tissues (i.e., roots and rhizomes) stabilize sediments to prevent erosion (Hemminga and Duarte 2000; Beer et al. 2014). Seagrass meadows represent a significant fraction of the global carbon stock (Fourqurean et al. 2012) and are essential for carbon cycling in coastal areas, as their photosynthesis and respiration strongly influence carbon budgets, especially in highly productive shallow-water areas (Buapet et al. 2013a). Their net primary production represents approximately 1% of total net marine primary production (Duarte and Cebrián 1996) and seagrass meadows have been identified as target ecosystems to be used when designing strategies to mitigate climate change (Duarte et al. 2013). Nevertheless, most estimates of net primary production may be inaccurate, because they do not consider that respiration in the plant may vary both with light (Rasmusson and Björk 2014) and on a diel basis (Procaccini et al. 2017). Most respiration values, in fact, are obtained during darkness and considered constant (Middelburg et al. 2005; Barron et al. 2006; Duarte et al. 2010). Although the calculated carbon capture rates are high, correcting the respiratory rate for diel fluctuations may modify the known net primary productivity, giving a more realistic estimate of seagrass habitats as marine carbon sinks.

Plant respiration is highly variable and sensitive to environmental cues such as temperature, light availability, and nutrient status (Marsh et al. 1986; Ryan 1991; Atkin and Tjoelker 2003; Millar et al. 2011), which allow plants to rapidly adapt to changes in the environment. In a fluctuating light environment, plants acclimate by adjusting their mitochondrial respiratory system (Yoshida et al. 2011). Reduced rates of mitochondrial respiration in light compared to darkness have been detected in numerous terrestrial plant species (Brooks and Farquhar 1985; Atkin et al. 2000; Hurry et al. 2005; Tcherkez et al. 2012) and have also been observed in *Z. marina* (Rasmusson and Björk 2014). This repression is widely discussed in the literature and it is proposed that either the suppression of the mitochondrial pyruvate dehydrogenase complex (mtPDC) or the inhibition of isocitrate dehydrogenase (IDH) can slow down the tricarboxylic acid (TCA) cycle (Budde and Randall 1990; Gemel and Randall 1992; Igamberdiev and Gardeström 2003; Tovar-Méndez et al. 2003). In addition, access to substrates (i.e., carbohydrates) alters plant respiration and is thought to be a major factor on the regulation of this process (Edwards and Mclaughlin 1978; Journet et al. 1986; Bunce 2001; Gary et al. 2003). Several plant function mechanisms, including respiration, are also influenced by circadian rhythms (McClung 2001; Dodd et al. 2005; Seaton et al. 2014), which might explain observed diel fluctuations.

The study of seagrass primary productivity can be approached in various ways, each having specific advantages and shortcomings. Direct measurements of photosynthetic and respiratory processes have traditionally been made using various gas exchange techniques (Zimmerman et al. 1989; Beer et al. 2001; Greve et al. 2003; Buapet et al. 2013b) and PAM fluorometry (Beer and Björk 2000; Silva and Santos 2004; Beer et al. 2014). Nevertheless, a combination of methods can help to obtain a more comprehensive understanding while approaching mechanisms and processes at various levels (Procaccini et al. 2012), as demonstrated by Mazzuca et al. (2013) and Procaccini et al. (2017). Gene regulation, as influenced by environmental variables such as light availability, depth, temperature, pH, and salinity, has been studied in *Z. marina* and the Mediterranean seagrass *Posidonia oceanica* by targeting gene RT-qPCR or RNA-Seq approaches (Reusch et al. 2008; Bergmann et al. 2010; Franssen et al. 2011; Winters et al. 2011; Serra et al. 2012; Dattolo et al. 2013, 2014; Marín-Guirao et al. 2016; Olivé et al. 2017). Attempts to correlate gene expression of the photosynthetic apparatus with productivity measurements (e.g., PAM fluorometry) have been successful (Winters et al. 2011; Mazzuca et al. 2013; Dattolo et al. 2014; Marín-Guirao et al. 2016); however, the regulation of respiration at transcript level has rarely been studied in seagrasses.

**Fig. 1:**
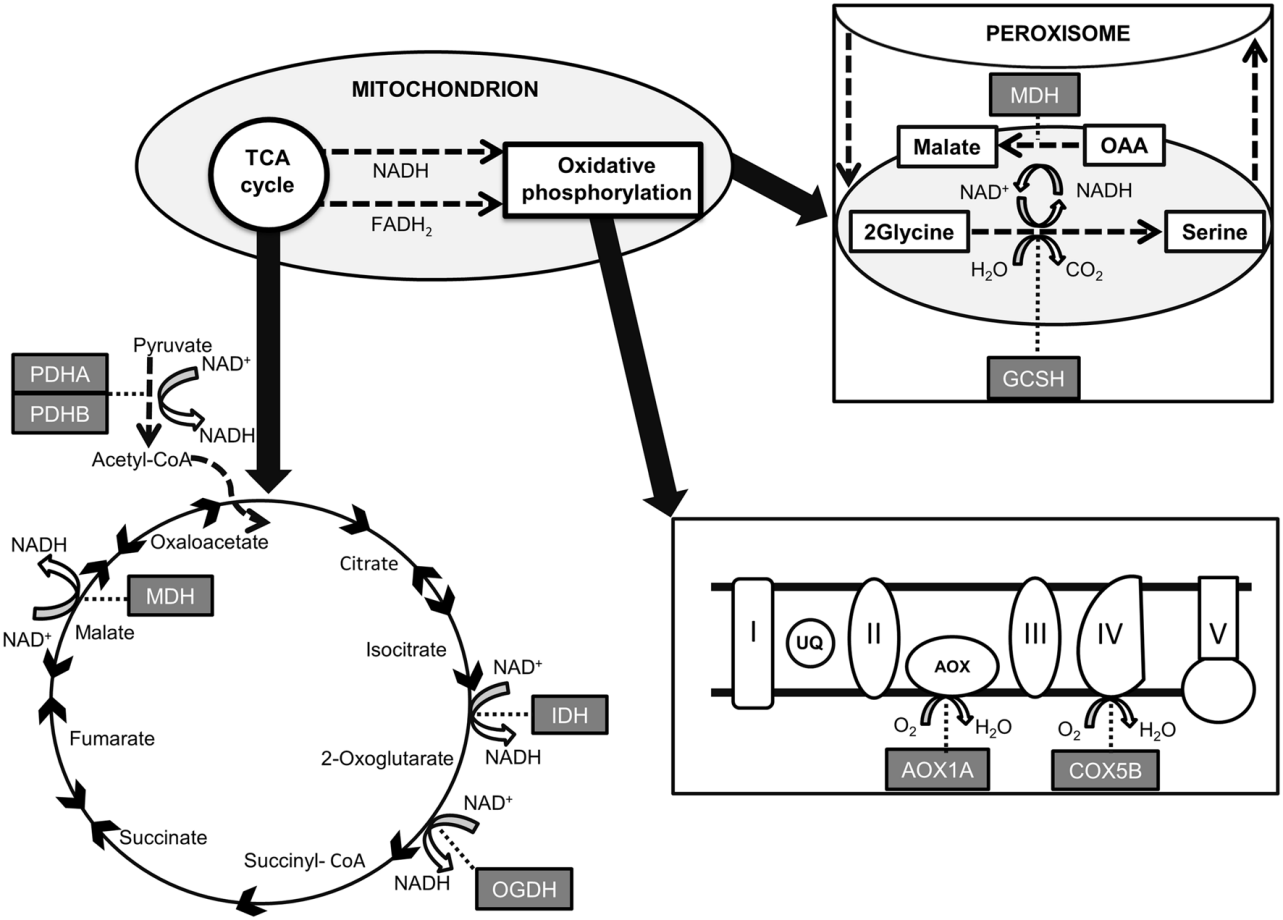
Highly simplified overview of various functions of the mitochondrion, adjusted to highlight the steps catalyzed by enzymes for which the respective encoding genes were studied in this work. *PDHA* and *PDHB*, situated in the mitochondrial matrix, catalyze the conversion of pyruvate into acetyl CoA, which enters the TCA cycle. Therein *IDH*, *OGDH*, and *MDH* all have decarboxylative functions generating NADH and releasing CO_2_. Note the *reverse arrow* between malate and oxaloacetate (OAA), in the *upper right corner* of the figure, indicating the adverse function of *MDH*, whereby NADH is instead oxidised into NAD^+^ used in the photorespiratory conversion of glycine into serine. This process is linked to the glycine cleavage system (GCS), in which the *GCSH* protein plays a crucial role. In oxidative phosphorylation (*lower right part* of the figure), electrons are transferred from reductants to O_2_ along the mitochondrial electron transport chain, consisting of transport proteins I–V, producing ATP. *COX5B* is the terminal oxidase in this chain, so the activity of this enzyme greatly influences ATP production. *AOX1A* is the terminal oxidase of the alternative pathway, which may protect the chain from electron overflow; however, the alternative pathway also lowers the yield of the chain, since less ATP is produced

This study assessed the role of natural light cycles in daily respiratory fluctuation in the temperate seagrass *Zostera marina*. To do this, we recorded oxygen consumption, measured by gas exchange techniques, at various time points, both in a field study and in controlled laboratory conditions. First, we performed field measurements where plants were taken instantly from the sea to the laboratory for measurements of respiration. Second, in a laboratory setup, we compared the oxygen consumption between plants exposed to natural daily summer light fluctuation and plants kept in the dark. In the latter, we also estimated changes in the expression of selected genes involved in the respiratory pathways (Fig. 1). Based on light-inhibited respiration measured in several terrestrial plant studies (Brooks and Farquhar 1985; Atkin et al. 2000; Hurry et al. 2005; Tcherkez et al. 2012) and in *Z. marina* (Rasmusson and Björk 2014), we hypothesized that lower respiration rates would be observed in leaves sampled during daylight. We were expecting an up-regulation of the genes related to the TCA cycle, i.e., pyruvate dehydrogenase (*PDHA* and *PDHB*), malate dehydrogenase (*MDH*), 2-oxoglutarate dehydrogenase (*OGDH*) and *IDH,* and the final oxidase of the mitochondrial electron chain, cytochrome C subunit 5b (*COX5B*), with higher respiration. Higher light conditions were also expected to decrease the transcript abundance of *PDHA, PDHB,* and *IDH*, as down-regulation of these genes in light has previously been reported (Budde and Randall 1990; Gemel and Randall 1992; Igamberdiev and Gardeström 2003). Up-regulation in high light was expected for the alternative oxidase (*AOX1*), as this may protect the mitochondrial electron chain from over excitation (Maxwell et al. 1999; Clifton et al. 2006), and glycine cleavage system H (*GCSH*) as an indication of increased photorespiration (Douce et al. 2001).

## Materials and methods

### Study site and plant material

**Fig. 2:**
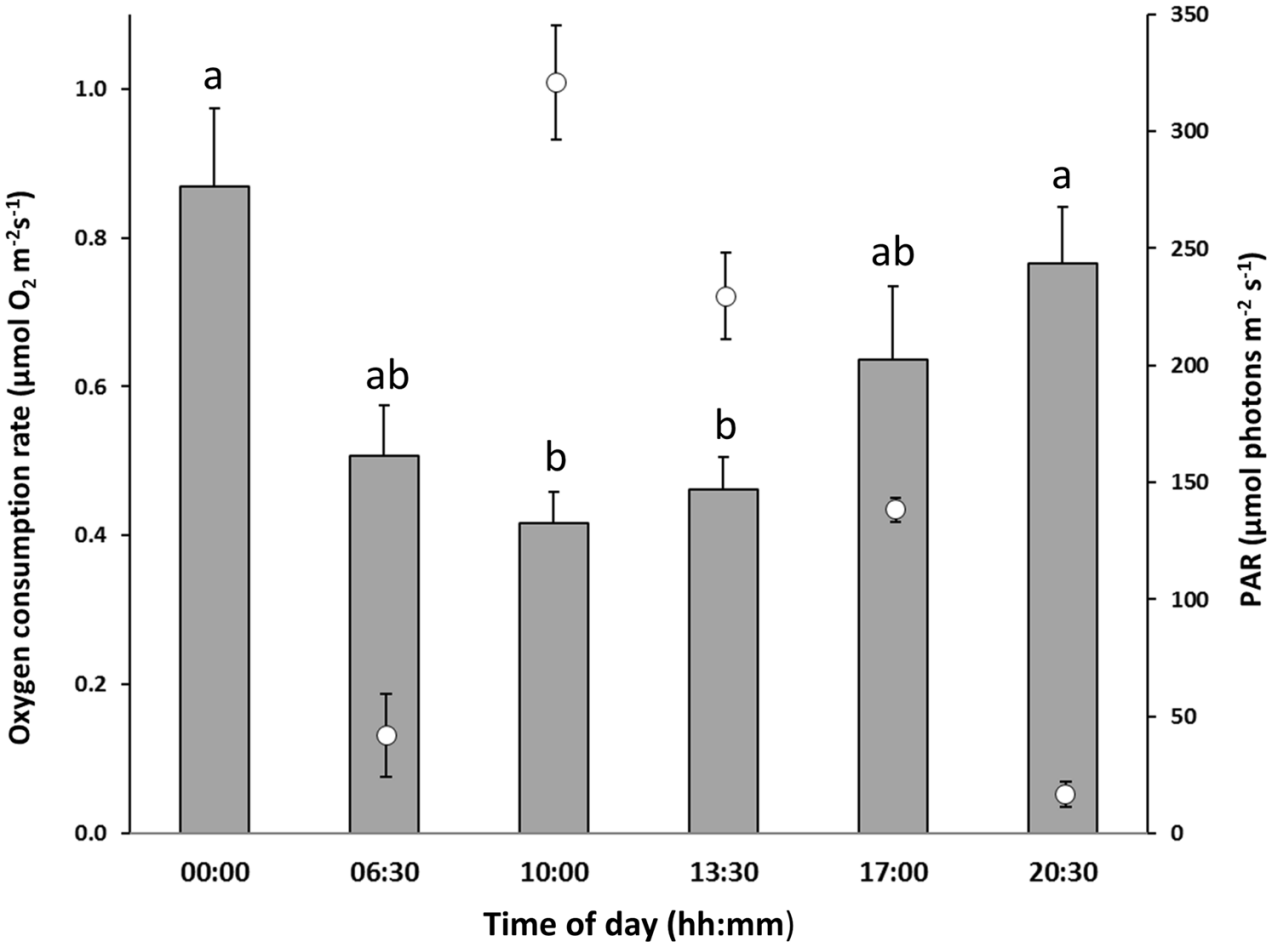
Oxygen consumption in the seagrass samples at the different time points in the field study. *Each bar* represents a mean value of 9–15 replicates ± SE. The variability in number of replications is due to problems with oxygen electrodes not functioning or seagrass specimens of poor quality. *Letters a* and *b* above *error bars* indicate significant differences (ANOVA, *P* < 0.05) between time points. The *white circles* represent a mean of the photosynthetic active radiation (PAR) measured in the seagrass meadow at the specific time point (*n* = 3) ± SE

The gas exchange measurements and collection of plants for the gene expression study took place at the Sven Lovén Centre for Marine Sciences (SLC), Kristineberg, Fiskebäckskil, on the Swedish west coast in July 2012 (field study) and June 2013 (laboratory study). The gene expression analyses were conducted at Stazione Zoologica Anton Dohrn (SZN), Naples, Italy, in November and December 2013. Shoots of *Z. marina* were collected in a healthy seagrass meadow, at approximately 2 m depth, located just outside the dock of SLC (58°15′0.13″N 11°26′46.95″O). For the field study, a minimum of three shoots were collected just before each measurement at the site and taken instantly (~10 min) to the laboratory for gas exchange measurements. Measurements and sampling were carried out at 06:30 (for three of the days), 10:00 (4 days), 13:30 (4 days), 17:00 (5 days), 20:30 (5 days), and 00:00 (3 days). All days of measurement had similar weather and light conditions. The uneven number of measurement days is due to logistical matters and weather conditions. The PAR irradiances at the collection site were estimated using a Diving-PAM (Walz, Effeltrich, Germany), at the time points of measurements (Fig. 2). For the laboratory study, rhizomes with up to three shoots were carefully collected at the site, transported within 10 min in a water-filled bucket, and planted in aquaria in the laboratory. Specimens were allowed to acclimate for 1 week before the experiment started. Three replicate aquaria were used, each with an approximate volume of 50 L and containing approximately 70 seagrass shoots. Sandy sediments from the seagrass meadow location were used as the planting substrate (approximately 8 cm in depth). Water was pumped directly from the ocean into the aquaria flow-through system, so levels of salinity (~24), oxygen concentration, pH, and temperature all followed the natural fluctuations over the day. Throughout the experiment, temperature, pH, and oxygen concentration were monitored in the aquaria (Table ESM1). Oxygen and temperature were monitored using dissolved oxygen data loggers (HOBO U26; Onset, Bourne, MA, USA) and pH was checked at every time point using a multimeter (pH probe: SenTix 940 connected to WTWMulti3430; WTW, Weilheim, Germany). Light was provided by luminous tubes (four tubes per aquaria) with cold white light (Aura Luminett 36 W; Aura Light, Stockholm, Sweden) and the intensity was altered during the day. The following PAR irradiances (µmol photons m^−2^ s^−1^) were used in sequence (the duration of each irradiance is indicated in parentheses): 20 (1 h), 40 (1 h), 70 (2 h), 120 (2 h), 150 (4 h), 70 (2 h), 40 (1 h), and 20 (2 h), the light being turned on at 06:30 and turned off at 21:30 (15 h:9 h, light:dark). These light cycles were used for the first well over 5 days (“light”), followed by well over 3 days of complete darkness (“continuous darkness”), to detect the presence of a potential endogenous circadian rhythm that could cause variations in respiration in the absence of external triggers (this setup is commonly used, e.g., Pallas et al. 1974; Hagemeyer and Waisel 1990; Sorek et al. 2013). Leaves for O_2_ measurements and RNA extraction were collected at 06:30, 10:00, 13:30, 17:00, 20:30, 00:00, and 03:15 (Table ESM2). “Light” measurements were initiated at 20:30 on day 1; in the evening of the seventh day, the aquaria were covered and kept dark for the remainder of the experiment. The first “continuous darkness” measurement was made at 06:30 the following morning. The experiment was terminated after 3 days in darkness and 10 days in total (Table ESM2). At each measurement, two or three shoots were randomly chosen from the aquaria (Table ESM2). The second-youngest leaf was removed from each shoot for the gas exchange measurements, and at the same time, a leaf segment of approximately 8 cm (removing apex- and basal parts) taken from the third-youngest leaf of the same shoot was cleaned with fresh water, fixed in RNAlater (Life Technologies, Carlsbad, CA, USA), and kept frozen at approximately −20 °C for future gene expression analyses. The rest of the shoot was left in the aquarium to maintain the biomass balance during the experiment.

### Gas exchange measurements

Clark-type oxygen electrodes (S1; Hansatech Instruments, King’s Lynn, UK) were used to determine oxygen fluctuations in the water in 3-mL electrode chambers (DW1/AD; Hansatech Instruments). Magnetic stirrers ensured constant water movement in the chambers. Natural seawater used had equilibrated oxygen levels, a salinity of approximately 23, and a pH of 8.10–8.26. The chamber was kept at ambient seawater temperature, as seawater directly from the ocean circulated into the water jacket surrounding the chamber. In the chamber, a leaf segment 3 cm long and 0.4–1 cm wide was fixed in a U shape. All leaves used were free of visible epiphytes. All oxygen measurements were carried out in darkness to eliminate influences of photosynthesis and/or photorespiration. Oxygen exchange was measured during 20 min of darkness and the value when a stable rate was reached was recorded (after approximately 10 min). This elapsed time prevented the influence of light-enhanced dark respiration (Heichel 1971; Raghavendra et al. 1994; Atkin et al. 1998), i.e., the peak of post-illumination oxygen consumption occurring after approximately 180–240 s. Oxygen uptake rates were calculated based on leaf area (one-sided).

### Gene expression analyses

#### RNA extraction and cDNA synthesis

Portions of the third-youngest leaf tissue were ground into a fine powder with a mortar and pestle using liquid nitrogen. For each time point, specimens from three different days were chosen for “light” and “continuous darkness”, respectively. Approximately 100 mg of powdered tissue was used for the RNA extraction using the AurumTM Total RNA Mini Kit (Bio-Rad Laboratories, Hercules, CA, USA) following the manufacturer’s instructions. After the lysis solution treatment, samples were homogenized using the Qiagen Tissue Lyser and tungsten carbide beads (3 mm) (Quiagen, Venlo, The Netherlands) for 3 min at 20.1 Hz. RNA quantity was determined using an ND-1000 UV–Vis spectrophotometer (NanoDrop Technologies, Wilmington, DE, USA) to monitor absorbance at 260 nm; purity was determined by monitoring the 260/280 and 260/230 nm ratios using the same instrument. RNA quality was evaluated using gel electrophoresis. RNA samples 500 ng/each were retrotranscribed into cDNA with the iScriptcDNA synthesis kit (Bio-Rad), following the manufacturer’s instructions, using the GeneAmp PCR System 9700 (Perkin Elmer, Waltham, MA, USA). The reaction was carried out as in Lauritano et al. (2015).

### Oligo design and PCR (polymerase chain reaction) optimization

Target genes were chosen based on their functions (Table 1). Primers for the target genes (Fig. 1; Table 1) were designed considering sequences from the seagrass EST database Dr. Zompo (Wissler et al. 2009) and from the generic online database GenBank (http://www.ncbi.nlm.nih.gov/genbank/). Primers were designed using the software Primer3 v. 0.4.0 (http://frodo.wi.mit.edu/primer3/) (Table 2) and optimized in a GeneAmp PCR System 9700 (Perkin Elmer). Reactions were carried out in a solution consisting of 2 µL of 10 × PCR reaction buffer (Roche, Basel, Switzerland), 2 µL of 0.1% BSA, 2 µL of 10 × 2 mM dNTP, 0.8 µL of 5 U µL^−1^ Taq (Roche), 1 µL of 20 pmol µL^−1^ for each oligo, 1 µL of template cDNA, and nuclease-free water added up to 20 µL. The PCR program consisted of a denaturation step at 95 °C for 3 min, 40 cycles at 95 °C for 30 s, 60 °C for 1 min, and 72 °C for 30 s, and a final extension step at 72 °C for 7 min. Amplified PCR products were analyzed by means of 1.5% agarose gel electrophoresis in TBE buffer. To verify the correct assignment of amplicons to target genes, the resulting bands were excised from the gel, extracted according to the QIAquick Gel Extraction Kit protocol (Qiagen), and the sequence analyzed. Sequence reactions were determined using Big Dye Terminator cycle sequencing technology (Applied Biosystems, Waltham, MA, USA) and purified using the Agencourt CleanSEQ Dye terminator removal kit (Agencourt Bioscience Corporation, Beverly, MA, USA), automated using the Biomek FX robotic station (Beckman Coulter, Brea, CA, USA). Products were analyzed on the Automated Capillary Electrophoresis Sequencer 3730 DNA Analyser (Applied Biosystems). Sequence identification was confirmed using the BLAST (Basic Local Alignment Search Tool) bioinformatics tool. Sequences were deposited in GenBank under the accession numbers shown in Table 2.

**Table 1:** Gene names, abbreviations, metabolic processes, and functions

Gene name	Abbreviations	Metabolic process	Gene function
Pyruvate dehydrogenase A	*PDHA*	Glycolysis	Decarboxylation of pyruvate to acetyl CoA; NAD^+^ reduction
Pyruvate dehydrogenase B	*PDHB*	Glycolysis	Decarboxylation of pyruvate to acetyl CoA; NAD^+^ reduction
Isocitrate dehydrogenase	*IDH*	TCA cycle	Decarboxylation of isocitrate to 2-oxoglutarate; NAD^+^ reduction
2-Oxoglutarate	*OGDH*	TCA cycle	Decarboxylation of 2-oxoglutarate to succinyl-CoA; NAD^+^ reduction; involved in nitrate assimilation
Malate dehydrogenase	*MDH*	TCA cycle	Oxidation of malate-to-oxaloacetate (OAA) = NAD^+^ reduction; reduction of OAA to malate = NADH oxidation, which provides reductant for Gly-to-Ser conversion in photorespiration
Alternative oxidase 1A	*AOX1A*	Mitochondrial electron transport chain	Terminal oxidase in the alternative pathway of the mitochondrial electron transport chain; reduces O_2_ to H_2_O; protective role during overexcitement; hampers ATP production
Cytochrome C subunit 5B	*COX5B*	Mitochondrial electron transport chain	Terminal oxidase in the main pathway of the mitochondrial electron transport chain; reduces O_2_ to H_2_O
Glycine cleavage system H protein	*GCSH*	Photorespiration	Decarboxylation of glycine to serine

**Table 2:** Genbank accession numbers, primer sequences (forward and reverse), PCR efficiencies (*E*), and correlation factors (*R* ^2^) of the genes of interest

Abbreviations	Accession numbers	Forward	Reverse	*E*	*R* ^2^
*PDHA*	KP866138	GATGGGTGAAGAGGTTGGG	GATGATATGATCAATTGCCTGC	1.81	1
*PDHB*	KP866139	GGACACCTACAGATATCATGGTCA	CTTGGCAGTTGCAATAGCTTC	1.82	1
*IDH*	KP866143	CAGCTCAGAAAGGAGCTTGATC	GCATACTCAAATGCATACTTTGC	1.93	0.997
*OGDH*	KP866140	ACTGACAGTTTCCTTGATGCTTC	TCATGCTTGGCCTTCATGTG	1.81	0.999
*MDH*	KP866144	GGCAACTCCTTCGACAAATG	AGCTCAGTTACCGTTGACTGAAC	1.81	0.999
*AOX1A*	KP866137	CAGTTCCAGGTATGGTCGGA	GCGTTGAAGAACACACCTTGA	1.87	1
*COX5B*	KP866141	CCGATTGCTACTACTGGACACGA	CAGCCAAAACCAAACAACATC	1.8	0.999
*GCSH*	KP866142	TCTCATGAATGGGTCAAGCC	TGTAGCTTTAACACTCTCCACAGC	1.85	0.999

Key to abbreviated gene names is found in Table 1

### Reverse transcription-quantitative polymerase chain reaction (RT-qPCR)

Serial dilutions of cDNA were used to determine the gene primer reaction efficiency and correlation factor (Table 2), generating standard curves with five dilution points using the cycle threshold (Ct) value versus the logarithm of each dilution factor and using the equation *E* = 10^^1/slope^. We performed RT-qPCR in MicroAmp Optical 384-well reaction plates (Applied Biosystems) with optical adhesive covers (Applied Biosystems) in a Viia7 real-time PCR system (Applied Biosystems) and using the fluorescent dye Fast Start SYBR Green Master Mix (Roche). The PCR volume for each sample was 10 µL, with 5 µL of Fast Start SYBR Green Master Mix, 1 µL of cDNA template (1:50 template dilution), and 0.7 pmol mL^−1^ of each oligo. The RT-qPCR thermal profile was obtained using the following procedure: 95 °C for 20 s, 40 × 95 °C for 1 s, and 60 °C for 20 s. The program was set to reveal the melting curve of each amplicon from 60 to 95 °C, reading it every 0.5 °C. Only a single peak was identified in the melting-curve analyses of all genes, confirming gene-specific amplification and the absence of primer-dimers. All RT-qPCR reactions were carried out in triplicate to capture intra-assay variability. Each assay included three no-template negative controls for each primer pair. Expression levels were analyzed using the relative expression software tool (REST) (Pfaffl et al. 2002) with eukaryotic initiation factor 4A (*eIF4A*) as the reference gene (Ransbotyn and Reusch 2006; Bergmann et al. 2010; Winters et al. 2011). Results were normalized to the baseline level of the plants collected at midnight (00:00) from each of the two treatments.

### Statistical analyses

Based on data from the field study, one-way ANOVAs were used to test for differences in mean respiration rate among different time points and for differences among days within each time point. Based on data from the laboratory study, we performed a two-way ANOVA to test for differences in mean respiration rate using light condition (two levels) and time point (seven levels) as fixed factors. A one-way ANOVA was performed to test for differences among days within each time point. To identify time points (within days) that were significantly different from each other, a Tukey HSD post hoc test was performed. Prior to ANOVA analyses, data were tested for homogeneity of variances using Levene’s test (Levene 1960). Where homogeneity was not met, the data were log_10_(*x* + 1)-transformed. In the laboratory study, Pearson’s correlation coefficient was used to determine whether respiration was correlated with pH, oxygen, and/or temperature, respectively. Statistica v. 13 software was used for all ANOVAs and Pearson’s correlations tests. For the gene expression data, unpaired *t* tests, corresponding to the difference in expression level of each gene compared to its corresponding control, were performed, using both the Randomization test from REST (Pfaffl et al. 2002) and GraphPad Prim V4.00 statistical software (GraphPad Software, La Jolla, CA, USA). The significance level for all analyses was set at *P* < 0.05.

## Results

### Gas exchange analyses

Oxygen consumption in samples collected in the field was significantly lower earlier in the day (i.e., 10:00 and 13:30) than in the evening and at night (i.e., 20:30 and 00:00) (Fig. 2). 10:00 was different to 20:30 and 00:00 (ANOVA, *P* < 0.05 and *P* < 0.01, respectively) and 13:30–20:30 and 00:00 (ANOVA, *P* < 0.05 for both). At 06:30, lower (although not significant, ANOVA, *P* = 0.07) respiration rates were detected as well. Effect of which day within each time point the measurement was conducted showed significant differences at a few time points, including 10:00 (difference between day 3 and 6, ANOVA, *P* < 0.05), 13:30 (between day 4 and 5, ANOVA, *P* < 0.05 and between day 4 and 6, *P* < 0.05), and 17:00 (between day 3 and 5, ANOVA, *P* < 0.05 and between day 3 and 6, *P* < 0.05). As these differences were significant yet highly random, they will most likely not be of significance from a biological point of view and influence the results. We will thus not discuss this further.

**Fig. 3:**
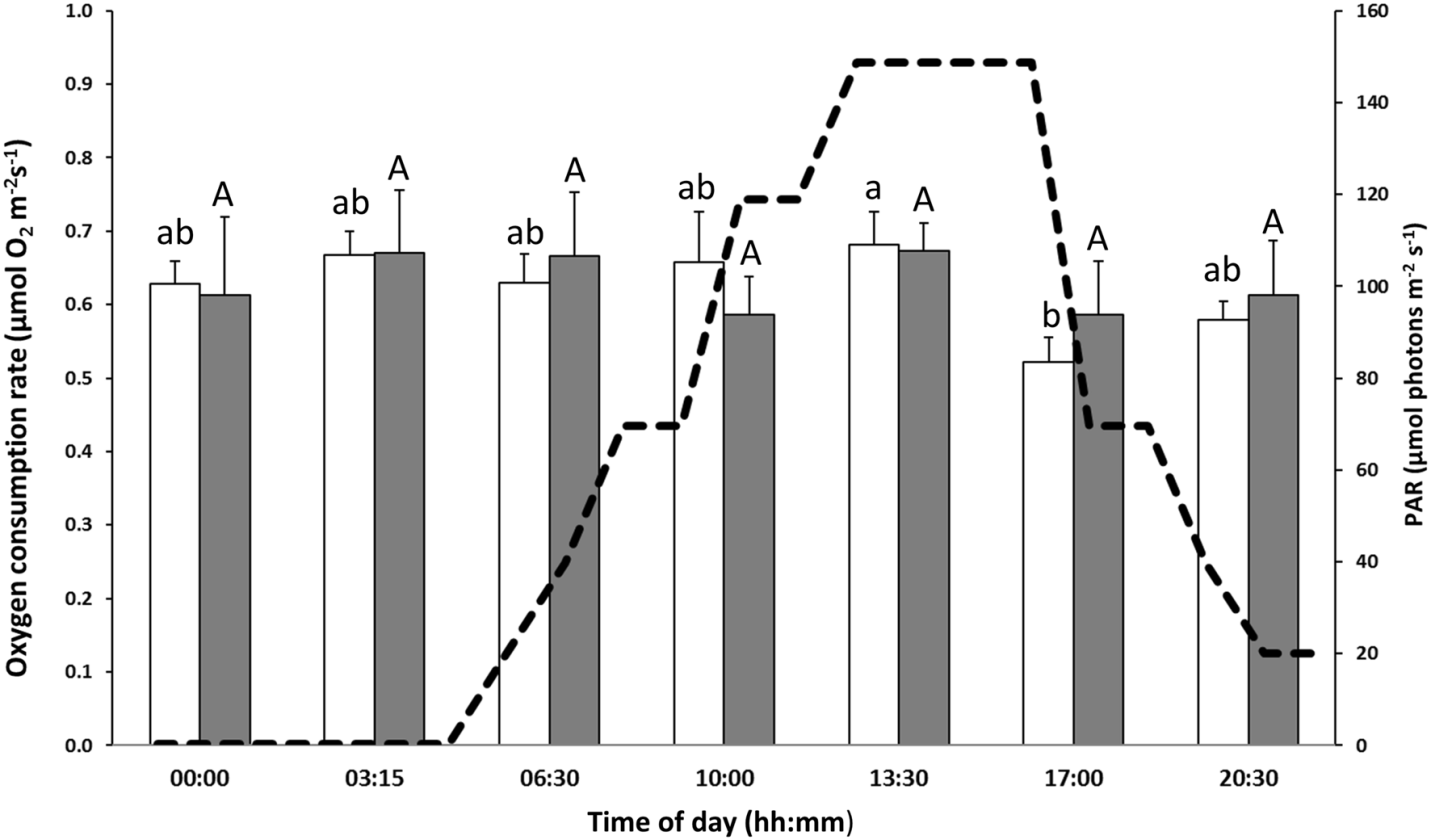
Rate of oxygen consumption in seagrass samples taken at different time points during a day–night cycle in the laboratory setup. *Light bars* seagrass growing in light conditions (*n* = 5–12, 5.5 consecutive days), *dark bars* seagrass growing in continuous darkness (*n* = 4–7, 3.5 consecutive days). The number of replication variability is depending on malfunction of oxygen electrodes or poor viability of seagrass specimens. Each point represents the mean ± SE. *Letters a*, *b*, and *A* above *error bars* indicate significant differences (ANOVA, *P* < 0.05) between time points. The *line* represents the light irradiances, i.e., photosynthetic active radiation (PAR), provided in the aquaria in the “light” settings

During the course of the incubation in the laboratory study, there was a slow linear increase in the respiration rate (*y* = −0.0617*x* − 0.1895, *R* ^2^ = 0.4546, *P* < 0.05) that was constant in both the light and dark periods. Since we were mainly focusing on the daily variations per se, the respiration data were transformed to compensate for this drift by considering variations as originating from the regression line. After recalculation of the results, so that variations are seen as deviations from the regression line, the oxygen consumption appeared to follow similar patterns in samples from both light and dark treatments (Fig. 3). In “light” conditions, respiration was significantly higher (ANOVA, *P* < 0.05) in the middle of the day (i.e. 13:30) than at 17:00, while values at the other time points were generally lower (although not significantly) than those of 13:30. No significant differences were found among any of the different time points during the days in continuous darkness, and no differences between light treatments were found. Moreover, it should be noted that the first measurements in continuous darkness could be influenced by the transition from “light” to complete darkness. Day of measurement seemed to be of insignificance as no differences were found, except at 20:30 in continuous darkness where a difference was found between day 4 and 5 (ANOVA, *P* < 0.05).

No relationship was observed between respiration rate and oxygen concentration (Fig. ESM1a) or temperature (Fig. ESM1b) in the experimental chambers. A significant relationship (Pearson correlation, *P* < 0.01) was present between pH and respiratory rate (Fig. ESM1c), likely due to respiratory CO_2_ release.

### Gene expression analyses

**Fig. 4:**
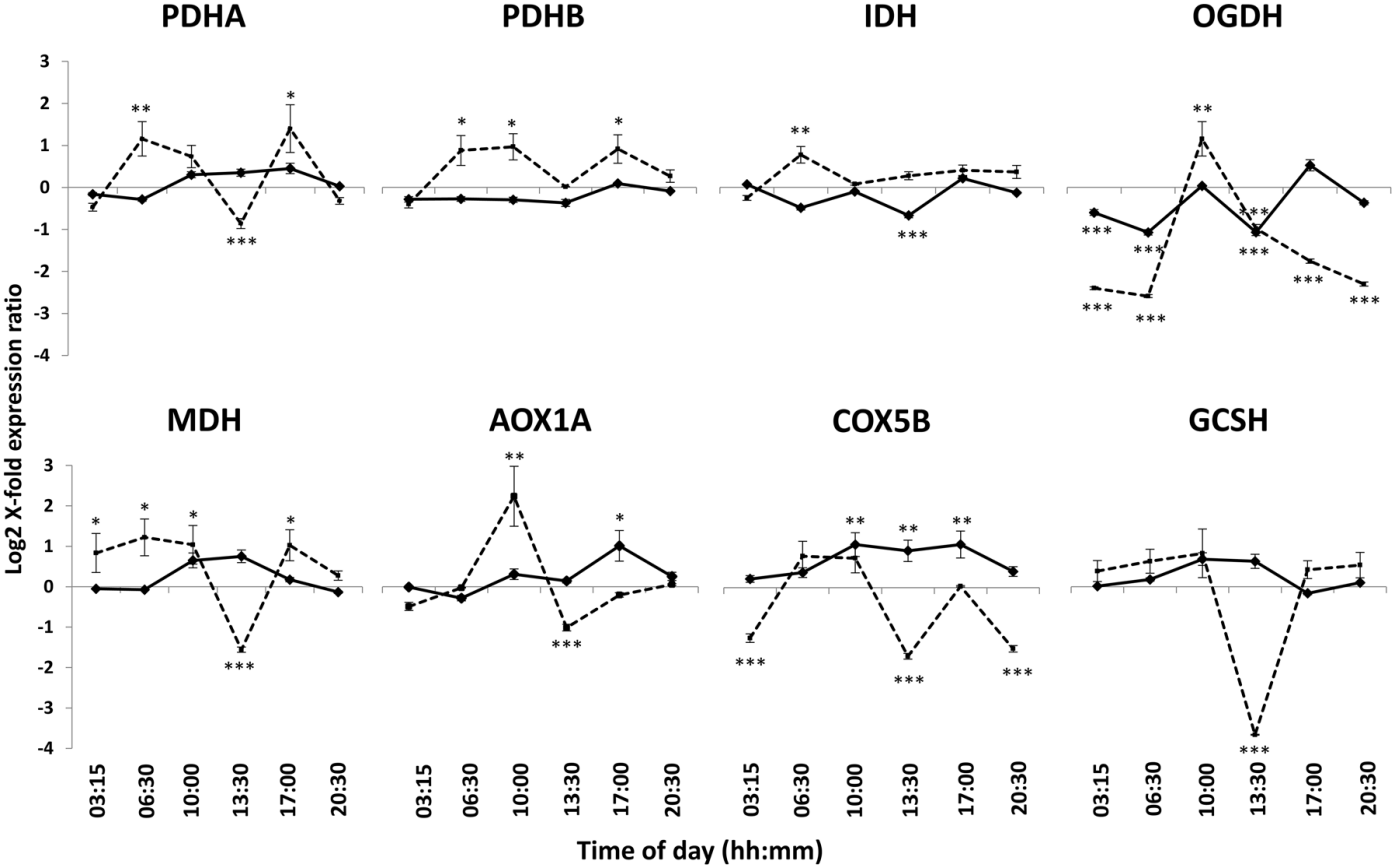
Expression level of the target genes in seagrass grown in conditions with light (*solid line*) and in continuous darkness (*dashed line*). Key to the abbreviated gene names is found in Table 1. Each value is a mean of three biological replicates and the results are presented with ± SD. All values have been normalized to the transcript abundance at midnight (00:00) from each treatment; the baseline level is thus different for “light” and “continuous darkness” samples. Significant differences between light- or dark-grown samples and the respective midnight baseline value are indicated as follows: *P < 0.05, **P < 0.01, and ***P < 0.001

All RNA extractions gave high RNA quality, with sharp ribosomal bands, as assessed using gel electrophoresis. All samples were free of proteins and organic solvents used during the RNA extraction, with 260/280 and 260/230 nm ratios always higher than 2.0. The relative transcript abundance of the target genes in samples taken at the various time points from plants grown in light and continuous darkness is shown in Fig. 4.

Among genes related to TCA cycle, two genes, i.e., isocitrate dehydrogenase (*IDH*) and 2-oxoglutarate dehydrogenase (*OGDH*), were down-regulated during light hours, with lower transcript abundance at 13:30, when the oxygen consumption rates were the highest (Fig. 3). *OGDH* had low expression rates also at 03:15 and 06:30 (Fig. 4). The third gene of the TCA cycle, malate dehydrogenase (*MDH*), together with the two subunits of pyruvate dehydrogenase (i.e., *PDHA* and *PDHB*), related to glycolysis, and the glycine cleavage system H (*GCSH*) showed no significant expression rates, indicating no strong influence of light on their transcript abundance. The two genes chosen from the mitochondrial electron transport chain, alternative oxidase subunit 1A (*AOX1A*) and cytochrome C subunit 5b (*COX5B*), were partly up-regulated at 17:00 (*AOX1A* and *COX5B*) and during the hours of high light (i.e., 10:00 and 13:30; *COX5B*).

Transcripts showed higher expression levels in plants kept in continuous darkness. A significant up-regulation was recorded at 06:30 for *PDHA*, *PDHB*, *IDH*, and *MDH* and at 10:00 for *PDHB*, *OGDH*, *MDH*, and *AOX1A*. At 13:30 all genes tested, except for *PDHB* and *IDH*, were down-regulated. Down-regulation of *OGDH* was also detected at all time points except 10:00. *COX5B* was down-regulated at 03:15 and 20:30.

## Discussion

This study showed that, in natural conditions, respiration in the temperate seagrass *Z. marina* varies on a diel basis. Diel variability of the oxygen consumption was basically absent in the laboratory setup under conditions mimicking natural light, and where water temperature, pH, and oxygen conditions were close to constant (due to minor fluctuations in the flow-through system). The laboratory results, therefore, suggest that light may not have as strong influence on the oxygen consumption as we expected and that the fluctuations were driven by other factors (e.g., fluctuations of temperature, oxygen, or pH). Nevertheless, the lower light intensity provided in the laboratory in the high light hours (i.e., 10:00 and 13:30) and also the difference in light quality between field and aquarium conditions may have influenced the results. As the slight diel variations of respiratory patterns in continuous darkness were not significant, we cannot unequivocally infer the presence of a circadian clock controlling the respiration of *Z. marina*.

In the field, respiration was lower in the morning and afternoon and increased towards the evening and night, when light was either absent or very low. The previous studies suggested a partial down-regulation of the TCA cycle in light, due to alterations of NADH and ADP in the mitochondrial matrix (Budde and Randall 1990; Gemel and Randall 1992; Tovar-Méndez et al. 2003). In accordance to this, two of the target genes linked to this process, *IDH* and *OGDH*, were down-regulated in high light at 13:30. In continuous darkness, the third target gene related to the TCA cycle (*MDH*) was up-regulated in the morning (at 06:30 and 10:00) and in the afternoon (at 17:00), something not detected when grown in the light, supporting the hypothesis of the inactivation of respiration upon illumination. Among the genes involved in the mitochondrial electron transport chain, only *COX5B* displayed a clear up-regulation in light. The alternative oxidase *AOX1A* is known to function under high light conditions, when it protects the machinery from overexcitement through the relocation of electrons and the formation of reactive oxygen species (Maxwell et al. 1999; Clifton et al. 2006). It showed, for instance, a clear light-dependent daily fluctuation in its expression in the seagrass *P. oceanica*, with higher values in the light hours (Procaccini et al. 2017). In our analysis, its significant over-expression only at 17:00, when ambient light was low in the aquaria, does not seem consistent with the expectations, and warrants further analysis.

The similar oxygen consumption rates for plants in light and continuous darkness and the more variable expression and higher transcript abundance under the condition of complete darkness were quite unexpected. Photosynthetically derived substrates (i.e., carbohydrates) necessary to satisfy respiratory demands should be depleted after prolonged darkness and lower respiration rates would be expected (Journet et al. 1986; Baysdorfer et al. 1988; Bunce 2001; Gary et al. 2003). Nevertheless, alternative mechanisms might be activated in the absence of light to provide necessary respiratory substrates. Other non-carbohydrate compounds (Brouquisse et al. 1991; Gary et al. 2003; Ishizaki et al. 2005) as well as stored carbohydrates from belowground tissues (i.e., roots and rhizomes) could be potential substrates supporting respiration, a strategy observed in *Z. marina*, which allows this species to maintain metabolic functions during winter (Zimmerman et al. 1989; Burke et al. 1996). The lower transcript abundance for most of the investigated genes at 13:30 is not in accordance with the results of the gas exchange analyses, in which the respiration rate instead increased slightly. It might be that changes of respiration take place at translational or post-translational levels and is, therefore, not seen as transcript abundance alterations (Lee et al. 2010) or that gene expressions are affected by factors other than light. Phosphorylation, for instance, can highly alter the activity of the TCA cycle in light as the mitochondrial pyruvate dehydrogenase complex is inactivated (Budde and Randall 1990; Gemel and Randall 1992; Tovar-Méndez et al. 2003), something that would not be detected on a transcript level. Alternatively, down-regulation of the pyruvate dehydrogenase gene could be due to a temporal mismatch between accumulation of transcripts and turnover of proteins. Proteins may be synthesized before the respiration peak (indicated by more up-regulated genes at 10:00 than at 13:30) and then degraded during high oxygen consumption at 13:30. After degradation, new proteins might be synthesized and regulation levels are yet again elevated, as indicated by some of the up-regulated genes at 17:00. Bläsing et al. (2005) showed that gene expressions in *Arabidopsis* are highly influenced by the availability of carbohydrates and not directly by light. Depletion of sugars in continuous darkness may, therefore, affect regulation of genes more strongly than in light.

Combining different methods for analyzing metabolic processes at different levels of functioning has provided more detailed information on seagrass eco-physiological performance (Mazzuca et al. 2013; Dattolo et al. 2014; Marín-Guirao et al. 2016; Procaccini et al. 2017). The two methods used here complement each other in evaluating the role of light in affecting respiration in *Z. marina.* Recent studies by Rheuban et al. (2014) and Adams et al. (2016) also discuss potential diel fluctuations of seagrass respiration, although in a meadow perspective. The higher rates which they encountered during day-time were attributed mainly to increase heterotrophic bacterial respiration (Rheuban et al. 2014; Adams et al. 2016), although altered respiration rates of the plants themselves were addressed as a potential source of variability (Adams et al. 2016). Diel respiratory fluctuations have also been shown for *P. oceanica* (Mazzuca et al. 2013; Procaccini et al. 2017). Although the oxygen consumption peaks for *P. oceanica* were not always concurrent with the *Z. marina* peaks found in our field study, *P. oceanica* also displayed a daily pattern with lowest respiration earlier in the day (i.e., from sunrise to 10.00 a.m.). This strongly suggests that respiration rates of seagrasses exhibit diel variation, although the drivers behind these fluctuations are not yet understood. That photosynthesis is varying substantially over the day and thus altering productivity patterns have previously been illustrated (Ralph et al. 1998; Silva and Santos 2003; Buapet et al. 2013a). However, since respiration has a significant impact on productivity estimations, the diel variation in the respiratory portion of primary productivity needs to be better understood. Our results show that the common way of calculating seagrass respiration (obtained in darkness) in carbon budgets as if it was a constant value is not correct. Depending on the time of the day, there could be drastic differences in dark respiration rates, as shown in the field study where the respiration rate went down with about 50% from midnight to 10:00 the following morning. Thus, if calculations are made from just one time point and considered constant over the day, there would be a great over- or under-estimation of the carbon dioxide release from respiration. As a further example, our highest respiration rate at 00:00 was 0.869 µmol O_2_ m^−2^ s^−1^, but when we calculated an average of the measured respiratory rates over a 24 h cycle, the mean was 0.642 µmol O_2_ m^−2^ s^−1^, showing that the use of only one time point could give an overestimation of respiratory rates of approximately 26%. These findings should, therefore, be considered in future studies when assessing carbon and oxygen fluxes for coastal vegetated marine habitats.

## Electronic supplementary material

Below is the link to the electronic supplementary material.

Supplementary material 1 (PDF 903 kb)
