# Supplementary material for: Respiratory oxygen consumption in the seagrass Zostera marina varies on a diel basis and is partly affected by light
Source: Mar Biol. 2017 May 27;164(6):140. doi: 10.1007/s00227-017-3168-z (PMC5446554; doi:10.1007/s00227-017-3168-z)
Supplement: Supplementary file 1 — Supplementary material 1 (PDF 903 kb) [file 227_2017_3168_MOESM1_ESM.pdf]

Electronic supplementary material

Respiratory oxygen consumption in the seagrass *Zostera marina* varies on a diel basis and is partly affected by light

Lina M. Rasmusson[1*], Chiara Lauritano[2], Gabriele Procaccini[2], Martin Gullström[1], Pimchanok Buapet[1,3], Mats Björk[1]

[1] Seagrass Ecology and Physiology Research Group, Department of Ecology, Environment and Plant Sciences, Stockholm University, SE-106 91 Stockholm, Sweden

[2] Department of Integrative Marine Ecology, Stazione Zoologica Anton Dohrn, Villa Comunale, 80121- Naples, Italy

[3] Department of Biology, Faculty of Science, Prince of Songkla University, Hat Yai 90112, Songkhla, Thailand

[*]Corresponding author: E-mail: lina.rasmusson@su.se

Table ESM1. Mean values of pH, oxygen concentrations and temperature in the aquaria at the time points when samples were taken for measurements (mean ± SD). Number of replicates in brackets.

Time	03:15	06:30	10:00	13:30	17:00	20:30	00:00
pH	8.15±0.00 (*n*=2)	8.13 ±0.06 (*n*=4)	8.18 ±0.04 (*n*=6)	8.19±0.04 (*n*=9)	8.25 ±0.03 (*n*=7)	8.21±0.03 (*n*=9)	8.20 ±0.04 (*n*=6)
O_2 (mg/L^{-1})	10.19±0.27 (*n*=5)	9.98 ±0.33 (*n*=4)	10.04±0.38 (*n*=4)	10.18±0.76 (*n*=5)	10.66±0.62 (*n*=5)	10.60±0.43 (*n*=5)	10.50±0.37 (*n*=5)
Temp (°C)	16.49±0.48 (*n*=6)	16.45±0.34 (*n*=5)	16.35±0.27 (*n*=6)	16.71±0.25 (*n*=7)	16.83±0.19 (*n*=6)	16.59±0.36 (*n*=7)	16.47±0.35 (*n*=7)

Table ESM2. Sampling scheme of the laboratory study with consecutive days (starting at 20:30 on day 1 and terminating at day 10 at 03:15) and time points. Light grey fields indicate measurements in "light" and dark grey in "continuous darkness". The number in brackets at each measuring occasion is the number of replicates, * is the occasions where samples were taken for gene expression analyses.

	1	2	3	4	5	6	7	8	9	10
**00:00**		(2) *		(2) *	(2) *		(3)	(1) *	(2) *	(1) *
**03:15**		(1) *	(2) *	(2) *		(2)		(2) *	(2) *	(2) *
**06:30**		(2) *	(2) *	(1) *			(3) *	(2) *	(2) *	
**10:00**		(2) *	(2) *	(2) *			(3) *	(2) *	(2) *	
**13:30**		(2) *	(2) *	(2) *	(2)	(3)	(3) *	(2) *	(2) *	
**17:00**		(2) *	(2) *	(2) *	(2)	(3)	(2) *	(2) *	(2) *	
**20:30**	(2)	(2) *	(2) *	(3) *	(3)		(2) *	(2) *	(1) *	

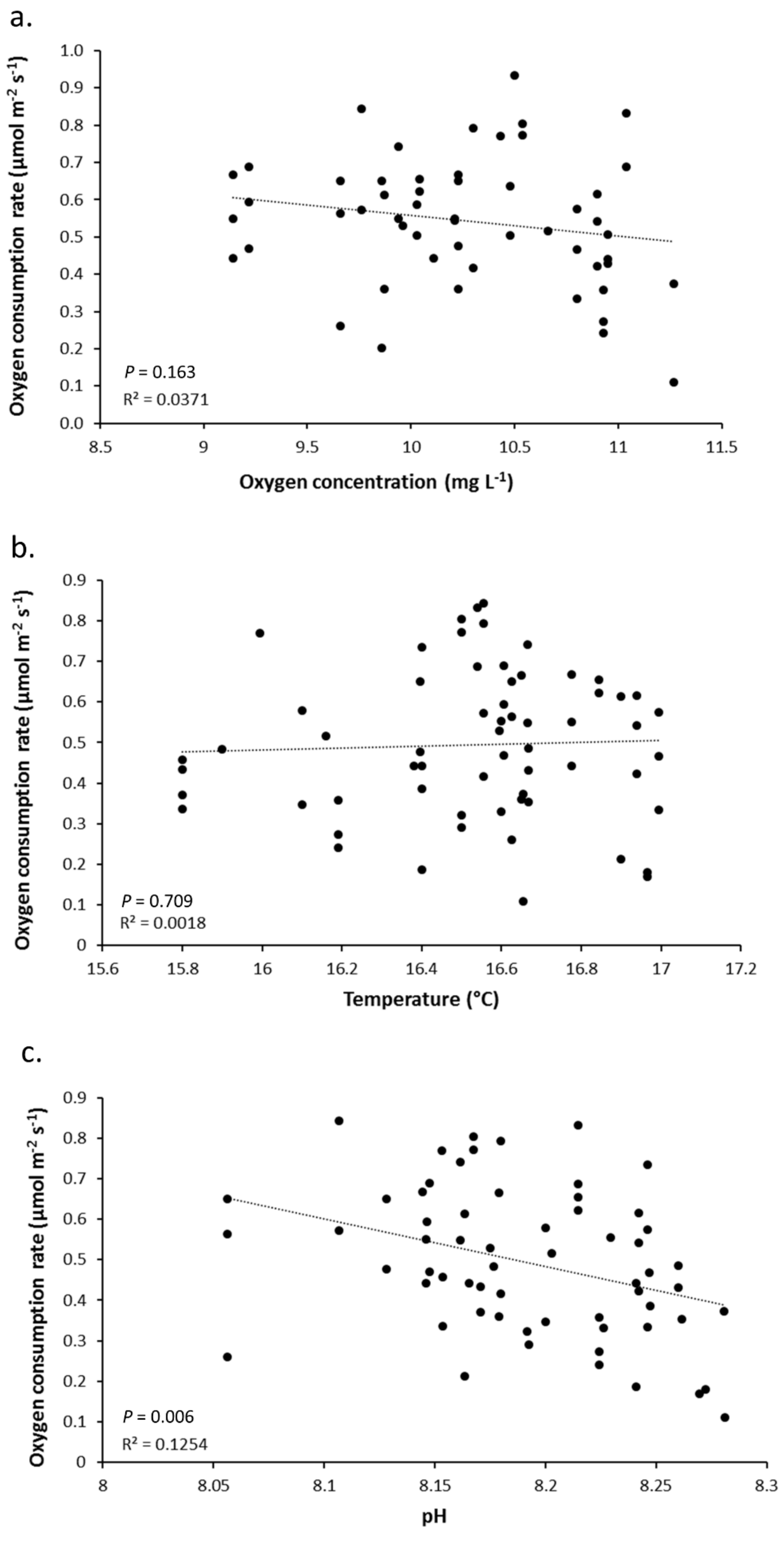

Fig. ESM1. The relationships between the oxygen consumption rate (y-axes) and oxygen concentration (a), temperature (b) and pH (c) in the experimental chambers. No relationship was observed between oxygen consumption rate and oxygen concentration or temperature. However, a clear relationship ($P < 0.01$) was present between oxygen consumption rate and pH
